# Turing Patterning Using Gene Circuits with Gas-Induced Degradation of Quorum Sensing Molecules

**DOI:** 10.1371/journal.pone.0153679

**Published:** 2016-05-05

**Authors:** Bartłomiej Borek, Jeff Hasty, Lev Tsimring

**Affiliations:** 1 BioCircuits Institute, University of California San Diego, 9500 Gilman Dr., La Jolla, CA, 92037-0328, United States of America; 2 San Diego Center for Systems Biology, University of California San Diego, 9500 Gilman Dr., La Jolla, CA, 92037-0375, United States of America; 3 Department of Bioengineering, University of California San Diego, 9500 Gilman Dr., La Jolla, CA, 92037-0412, United States of America; 4 Molecular Biology Section, Division of Biological Sciences, University of California San Diego, 9500 Gilman Dr., La Jolla, CA, 92037-0116, United States of America; Universitat Pompeu Fabra, SPAIN

## Abstract

The Turing instability was proposed more than six decades ago as a mechanism leading to spatial patterning, but it has yet to be exploited in a synthetic biology setting. Here we characterize the Turing instability in a specific gene circuit that can be implemented *in vitro* or in populations of clonal cells producing short-range activator N-Acyl homoserine lactone (AHL) and long-range inhibitor hydrogen peroxide (H_2_O_2_) gas. Slowing the production rate of the AHL-degrading enzyme, AiiA, generates stable fixed states, limit cycle oscillations and Turing patterns. Further tuning of signaling parameters determines local robustness and controls the range of unstable wavenumbers in the patterning regime. These findings provide a roadmap for optimizing spatial patterns of gene expression based on familiar quorum and gas sensitive *E. coli* promoters. The circuit design and predictions may be useful for (re)programming spatial dynamics in synthetic and natural gene expression systems.

## Introduction

Self-organization and self-assembly govern the emergent properties of spatial structures from the molecular to the galactic scale [1, 2]. In the nano-to-millimeter range chemical processes coordinate gene expression essential to the spatial organization of biological systems, including populations of microorganisms [3–6] and developing tissues [7–11].

One mechanism by which ensembles of cells could self-organize is the Turing instability [2, 12, 13] that occurs due to interplay of short-range activation and long-range inhibition. This instability then drives the formation of spatially periodic patterns. The Turing instability has been implicated in morphogenetic processes of amoebae [14], plants [15, 16], and animals [9, 10, 17–20]. The large number of unknown factors often makes it challenging to elucidate the essential determinants of morphogenesis in biological systems, but the instability has also been directly engineered in low-component chemical reactions (malonic acid [21] and platinum surface [22]).

Despite the ubiquity of Turing patterns at the multi-cellular scale, they have yet to be demonstrated in gene expression sytems. This is surprising given the many alternative pattern forming mechanisms found in natural [3, 4, 6] and engineered [5, 6, 23–27] colonies of cells. For example, researchers have created gene circuits to produce stationary ring patterns in growing colonies of bacteria [5, 24, 28]. However, these stationary patterns required colony expansion and very particular initial conditions to form. Hsia *e*t al. [29] have presented a theoretical model of a quenched oscillator circuit with one diffusible element that produces a Turing-like instability. There however, the temporal dynamics never settled to a stable fixed state due to persistent temporal oscillations. Another group [30] has recently developed a model of Turing pattern circuit using single promoter. However, even with common promoters, the activator and inhibitor genes would result in different production rate functions, especially if the compounds are to have largely differing diffusion rates. The lack of convincing examples raises the question of whether Turing patters can really be produced by genetic circuits.

If it is possible, then Turing patterning of gene expression could be quite useful for basic science and biotechnology applications. These include self-organized spatial sequestration of gene expression and downstream metabolites (as is currently done inside cells to improve yield [31]), spatially-patterned delivery of proteins [32], reprogrammed morphogenesis of cell populations [33], filtering of paracrine inputs from adjacent cells in populations [34], and chemical sensing [27]. Biosensing applications are particularly promising inasmuch as the Turing instability is inherently capable of amplifying small differences in initial conditions.

Motivated by this, we propose a realistic synthetic gene circuit that implements repressor-activated degradation using activating homoserine lactone and inhibitory peroxide gas, and demonstrate how this system can be switched between stable uniform states, limit cycles, and Turing patterns. We characterize the effects of experimentally relevant parameter changes on the presence and properties of the expected Turing patterns. We conclude by discussing our plans to experimentally implement the circuit and to refine the mathematical model that describes it.

## Methods

The fixed points, eigenvalues and unstable wavenumbers of the differential equations were computed using the symbolic and variable-precision solvers in Matlab [35]. Bifurcation analysis was performed using the Matcont continuation package [36], and analysis of the Turing instability was performed as stated in the text, with a custom Matlab script. Spatio-temporal simulations were performed using custom Fortran code [37] using forward Euler with five point discretized Laplacian. Convergence of solutions was validated by simulating successively smaller spatial and temporal step sizes. For most cases we also tested both periodic and no-flux boundary conditions and used dx = 11.5 *μ*m and dt = 10^−5^ min. We rescaled space by assuming *D*_*L*_ = 5.6 × 10^−3^ *μ*m^2^/min. S1 Table lists nominal parameter values used in the model equations.

## Results

### Turing pattern gene circuit with homoserine lactone and hydrogen peroxide gas

**Fig 1:**
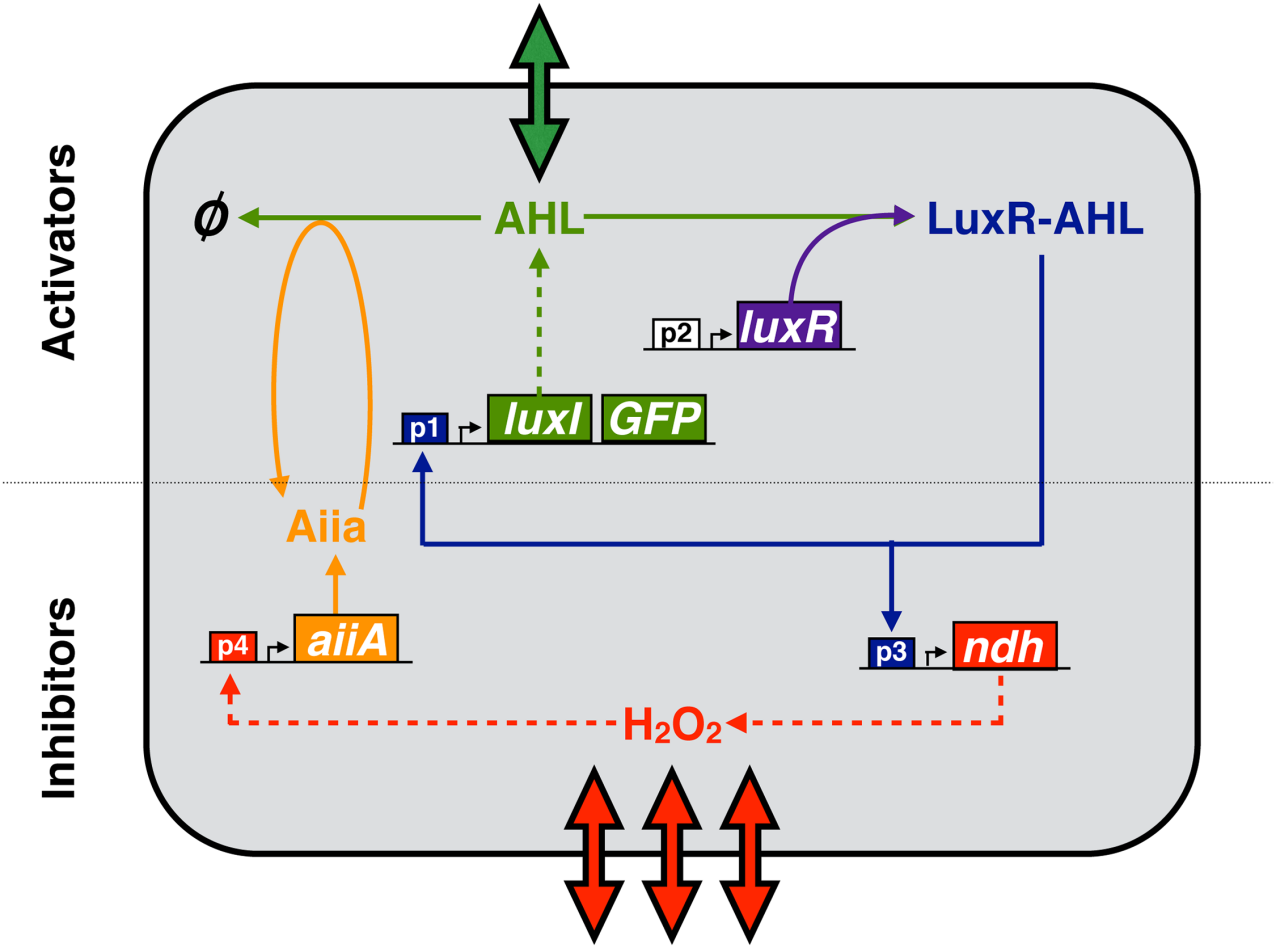
The proposed gene circuit for generation of Turing patterns. AHL activates its own production, but is degraded by Aiia. H_2_O_2_ is produced via the *ndh* gene, and activates the transcription of the *aiiA* gene. Intercellular transport and diffusion of AHL and H_2_O_2_ are represented by the thick arrows. The circuit is modeled by Eqs (1)–(4).

Based on our experience with using quorum-sensing signaling in synthetic circuit design [26, 27], we decided to use acyl homoserine lactone (AHL) as the short-range activator signal and hydrogen peroxide gas (H_2_O_2_) as the long-range inhibitor signal (Fig 1). The choice to focus on a pure activator-inhibitor system [38] (where the activator self-activates and inhibitor self-inhibits) was based on the fact that the AHL quorum sensing system has a native positive feedback, and H_2_O_2_ does not. Several of the circuit components presented in Fig 1 have already been used to construct gene circuits that produced sustained temporal oscillations of fluorescent proteins [27]. The synthase LuxI produces AHL, and the Ndh protein generates H_2_O_2_. AHL binds with LuxR and activates the p_*lux*_-like promoters (p_1_ and p_2_ in Fig 1). The *luxR* promoter (p_3_ in Fig 1) is constitutive (unregulated). Controlled degradation of AHL is mediated by the AHL-lactonase, AiiA [39]. The *aiiA* gene can be activated by H_2_O_2_ by putting it under the control of a promoter (p_4_ in Fig 1) such as: p_*topA*_, which responds to H_2_O_2_ via the Fis pathway [40], or a synthetic promoter containing the ArcAB binding site of p_*lux*_, which relieves inhibition by transcription factors ArcAB in the presence of the peroxide [41]).

The compound chemical reactions (transcription, translation, protein binding processing) underlying the proposed gene circuit are:

$$\begin{array}{ccc} & & \emptyset \overset{\alpha_{1}f_{1}(P)}{⟶}L\overset{\gamma_{1}g(L)}{⟶}\emptyset \\ & & \emptyset \overset{\alpha_{2}f_{2}(P)}{⟶}H\overset{\gamma_{2}}{⟶}\emptyset \\ & & \emptyset \overset{\alpha_{3}f_{3}(H)}{⟶}A\overset{\gamma_{3}}{⟶}\emptyset \\ & & L+L_{R}\overset{k_{1}}{⟶}P\overset{k_{2}}{⟶}L+L_{R} \end{array}$$

where *L*, *H*, *A*, *L*_*R*_, *P* represent AHL, H_2_O_2_, Aiia, LuxR and the AHL-LuxR complex, respectively. Production is controlled by *f*_*i*_(*Z*) = (*δ*_*i*_ + *Z*)/(*c*_*i*_ + *Z*), where *c*_1_ = *k*_*PL*_, *c*_2_ = *k*_*PH*_, *c*_3_ = *k*_*HA*_ are the thresholds for production of each species. Degradation is first order for H_2_O_2_ and Aiia, but enzymatic for AHL, with *g*(*L*) = *A*/(*k*_*LD*_ + *L*). Total LuxR is assumed to be constant, and the AHL-LuxR complex (with relatively fast association and dissociation rates *k*_1_, *k*_2_) is assumed to be relatively stable to degradation.

Including mass-action dynamics of these reactions along with diffusion, and rescaling *x* by ${D_{L}}^{-1/2}$ such that $D=\frac{D_{H}}{D_{L}}$, results in the following equations,

$$\begin{array}{c} \partial_{t}L=\alpha_{1}\frac{\delta_{1}+P}{k_{PL}+P}-\gamma_{1}A\frac{L}{k_{LD}+L}-k_{1}\left(P_{m}-P\right)L+k_{2}P+\nabla^{2}L \end{array}$$ (1)

$$\begin{array}{c} \partial_{t}P=k_{1}\left(P_{m}-P\right)L-k_{2}P \end{array}$$ (2)

$$\begin{array}{c} \partial_{t}H=\alpha_{2}\frac{\delta_{2}+P}{k_{PH}+P}-\gamma_{2}H+D\nabla^{2}H \end{array}$$ (3)

$$\begin{array}{c} \partial_{t}A=\alpha_{3}\frac{\delta_{3}+H}{k_{HA}+H}-\gamma_{3}A \end{array}$$ (4)

where *P*_*m*_ is the total amount of LuxR. The parameters of Eqs (1)–(4), and their nominal values used are listed in S1 Table.

### Reducing Degradation Feedback Generates Limit Cycles and Turing Patterns

**Fig 2:**
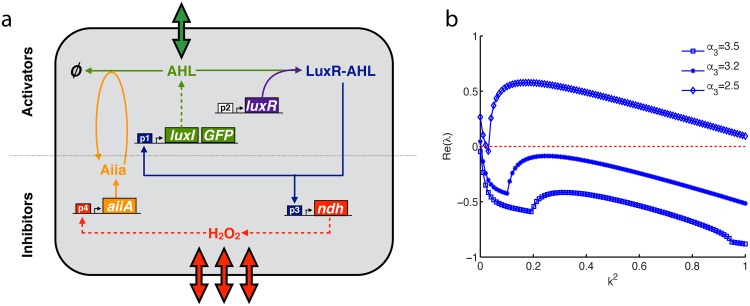
Slowing AiiA production in Eqs (1)–(4) leads to oscillations and Turing patterns. A codimension one bifurcation of the AHL fixed point, L_*_, losing stability through a Hopf bifurcation and into a Turing instability as Aiia production rate is decreased. (b) Eigenvalue-wavenumber curves at various AiiA maximal production rates, corroborating the bifurcation analysis results above. At the cusp of each curve the eigenvalues become a complex conjugate pair, with each low eigenvalue left off the plot for clarity.

Restricting AiiA dynamics to reasonable relative production and degradation rates [26, 27], we find parameter regimes of stable fixed points, limit cycles, and Turing patterning. The maximal Aiia production rate, *α*_3_, can be adjusted with plasmid copy number, ribosome binding site strength, or by using a hybrid promoter tuned with an inducer. Numerical analysis of the eigenvalues of the stable fixed point in Eqs (1)–(4) shows that slowing this maximal production rate leads to a stable limit cycle oscillation through a supercritical Hopf bifurcation, followed by a Turing instability, and a saddle node bifurcation (Fig 2a). The Turing instability bifurcation point is found by examining the characteristic equation,

$$\begin{array}{c} |J-\lambda I-k^{2}D|=0 \end{array}$$ (5)

for eigenvalues λ, where **J** is the Jacobian, **I** is the identity matrix, **D** is the matrix of diffusion coefficients, and *k* is the wavenumber. This equation is solved for different *α*_3_, with the largest positive real parts of the eigenvalues plotted in Fig 2b. Having only two diffusable substances leads to a quadratic equation in *k*^2^, *A*_(λ)_(*k*^2^)^2^ + *B*_(λ)_(*k*^2^) + *C*_(λ)_ = 0. Finding the root of the discriminant of the quadratic, $B_{\left(\lambda =0\right)}^{2}-4A_{\left(\lambda =0\right)}C_{\left(\lambda =0\right)}$, gives the condition for the Turing instability boundary (*α*_3_ = 3.1 min.^−1^ in Fig 2a)).

**Fig 3:**
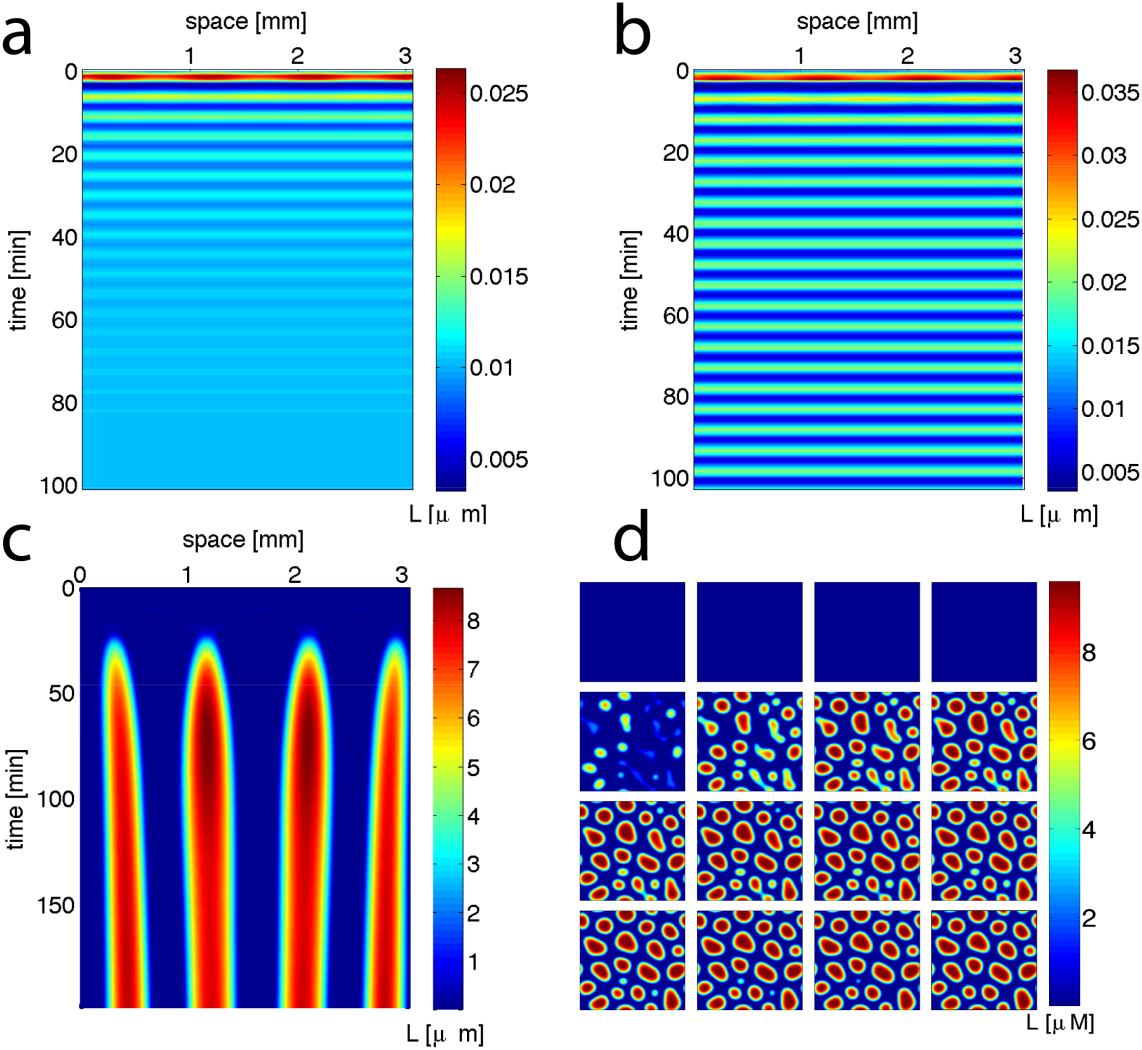
Numerical simulations of spatial dynamics in the model, Eqs (1)–(4), with periodic boundary conditions. (a-c) For 1-D space, slowing Aiia production confirms (a) stable focus at *α*_3_ = 3.5 min.^−1^, (b) limit cycle at *α*_3_ = 3.2 min.^−1^, and (c) Turing patterns at *α*_3_ = 2.5 min.^−1^. (d) The same parameters as (c) lead to spot patterns in 2-D simulations with periodic boundary conditions (4.87 × 4.87 cm^2^ panels taken every 13 mins).

Numerical simulations of the system in one spatial dimension with periodic boundary conditions further confirm a stable focus at *α*_3_ = 3.5 min.^−1^ (Fig 3a), a stable limit cycle at *α*_3_ = 3.2 min.^−1^ (Fig 3b), and Turing instability at *α*_3_ = 2.5 min.^−1^ (Fig 3c). In two spatial dimensions the instability leads to formation of spot patterns (for snapshots of simulations with periodic boundary conditions, see Fig 3d, and for movies with no-flux boundary conditions, see S1 Movie). Our observation of Turing instability taking over from Hopf instability has previously seen in other systems [42–44]. Lowering the AiiA degradation rate (or the slowing of AiiA dynamics in general) leads to a similar sequence of transitions from a homogeneous steady state to temporal oscillations followed by Turing patterns, but this was not investigated in detail, as such parameters would be more difficult to control experimentally.

Overall, the results indicate that slowing AiiA feedback on AHL degradation can lead to limit cycle oscillations before the onset of Turing patterns. This is consistent with previous studies showing that an increased time delay in a negative feedback signal produces limit cycle oscillations [45–47]. It is interesting to note that for the cases shown in Fig 2, the fixed point is already unstable at *k* = 0 (a limit cycle) when undergoing the transition to the Turing instability. This means that the classic notion of Turing patterns arising from a stable fixed point can be extended to include some cases with an unstable point inside a limit cycle.

### Controlling Range of Unstable Wavenumbers and Patterning Robustness

We next investigate how additional parameters can be used to experimentally tune spatial patterning in the circuit. In contrast to the single promoter circuit model that was proposed [30], increasing the cooperativity of transcriptional activation in our model does not appear to increase the parameter space leading to Turing patterns, since an increased rate of activation is not automatically balanced by an increased rate of repression. This however is not a major concern since increasing cooperativity would be challenging to implement in experiments.

**Fig 4:**
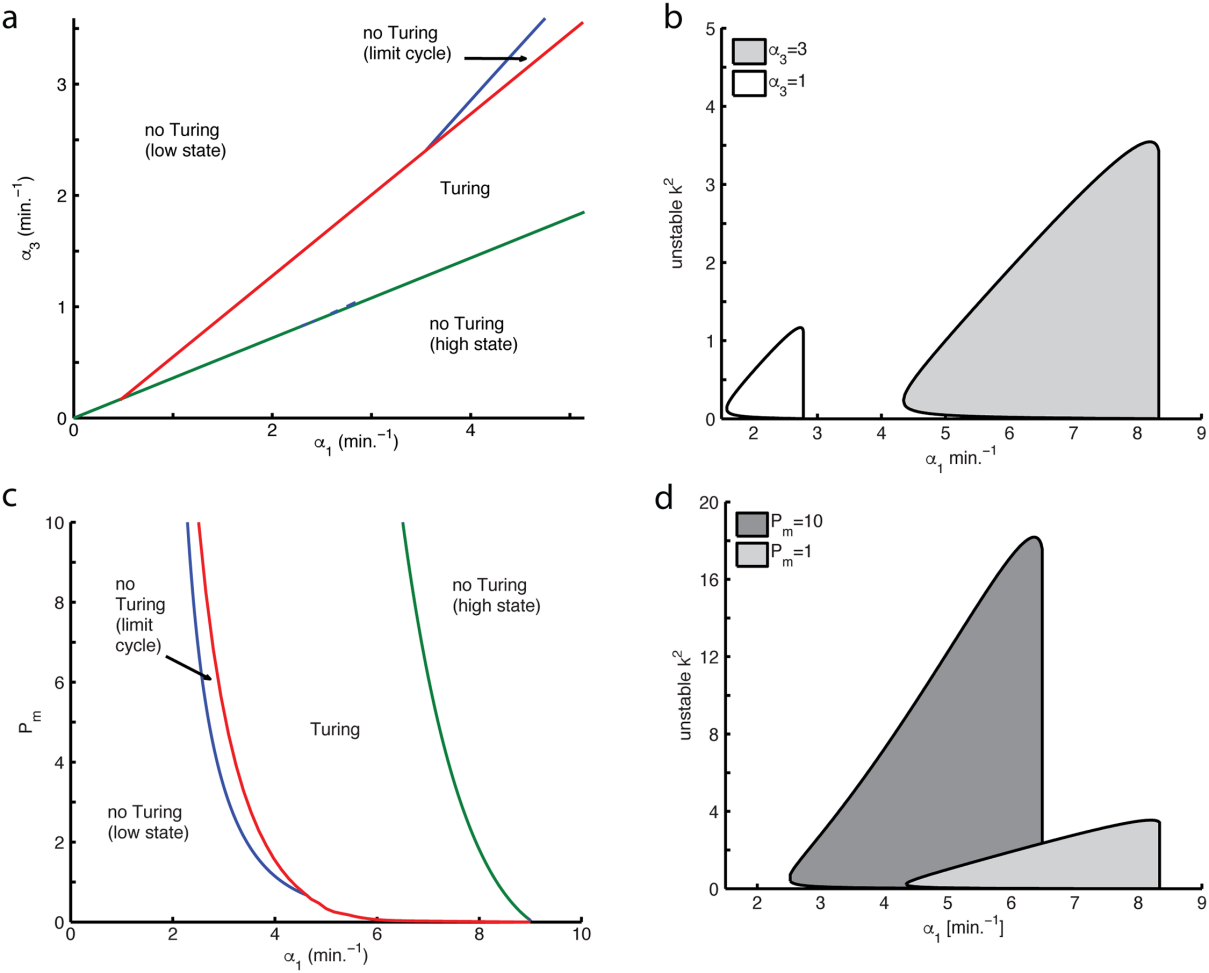
Dependence of Turing parameter space and wavenumbers on quorum sensing and AiiA parameters. (a,c) Two-parameter sections of Turing space, with curves of limit points (green), Hopf points (red), and Turing points (blue). (b,d) Ranges of Turing unstable wavenumbers for different sections along the maximal LuxI production rate within the Turing parameter space. Parameter values are varied from the nominal set (S1 Table).

A more experimentally tuneable parameter is *α*_1_, affecting the maximal rate of LuxI expression, and experimentally controlled by a regulatable hybrid promoter, plasmid copy number, or the strength of the promoter or ribosome binding site. Covarying this parameter with the AiiA production rate creates basins for stable, Hopf unstable and Turing unstable solutions (Fig 4a). For *α*_3_<0.16 min.^−1^ there is no *α*_1_ that gives rise to Turing patterning. For the parameter space investigated (Fig 4a) as *α*_1_ is increased, the range of *α*_3_ leading to Turing patterning (*α*_3_-robustness) also increases. Raising *α*_3_ also tends to increase *α*_1_-robustness (Fig 4a and 4b). Fig 4b demonstrates that raising the AiiA production rate expands the range of wavenumbers corresponding to the Turing instability, *Re*(λ(*k*)) > 0 towards higher wavenumbers (shorter wavelengths). Thus, tuning the maximal AiiA production rate to achieve more *α*_1_-robust Turing patterning also results in shorter spatial periods in the resultant pattern.

Another parameter to control experimentally is *P*_*m*_, representing the amount of LuxR. At the nominal parameter set (S1 Table), the Turing instability occurs for *P*_*m*_ > 0.18 (Fig 4c). Overall, the *P*_*m*_-robustness of Turing patterning is not very sensitive to *α*_1_, except near the borders of the Turing space (Fig 4c). Furthermore, the *α*_1_-robustness of patterning is not much affected by increasing *P*_*m*_ (Fig 4c and 4d). It is clear however that a higher amount of LuxR leads to much shorter spatial periods in the Turing pattern (Fig 4d). Both codimension two bifurcation plots (Fig 4a and 4c) show that the Turing regime can be adjacent in parameter space to a regime of limit cycle dynamics (as in Fig 2), but this is not always the case (for example, decreasing *α*_1_ when 0.5 < *α*_3_ < 3.2, in Fig 4a).

Together these results show that Turing patterning with this circuit requires an AHL production rate high enough to prevent a stable focus or limit cycle, but not so high as to lose the fixed point through a saddle node bifurcation. Furthermore, it appears that the AiiA production rate is the highest-priority parameter to adjust in order to achieve Turing patterning that is robust to variations in the other experimental parameters. Once inside the Turing unstable regime, both LuxI production rate and the amount of LuxR can be used to tune the range of spatial periods in the solution.

## Discussion

In this paper we have demonstrated the possibility of generating Turing patterns using a long-range gas signal that induces degradation of a short-range quorum sensing molecules. The next important step is to experimentally implement this proposed circuit in and test the predictions made by the model. The gene circuit has features in common with previous designs [26, 27], but there are some important differences. The Aiia promoter would likely need to be redesigned to be activated by exclusively by H_2_O_2_ instead of LuxR-AHL. Besides the use of *ndh*, H_2_O_2_ generation could also be increased by addition of a *lux*-induced *sodA* gene [27]. For the *lux*-like promoters (p_1_ and p_3_ in Fig 1), the model shows that they should be distinct from each other, with leak and maximal production from p_1_ being higher than those from p_3_ (compare *δ*_1,2_’s and *α*_1,2_’s in S1 Table). Alternatively, this could be achieved by putting ribosome binding sites on *luxI* that are stronger than those on *ndh*. It may also be necessary to remove the ArcAB sites on these promoters to eliminate crosstalk with the H_2_O_2_ part of the circuit, but preliminary analysis shows that crosstalk as high as 20% can still lead to patterning (see S1 Equations) and S3 Fig. One could also consider using alternative quorum sensing systems [48] and volatile signaling molecules such as nitric oxide [49], ammonia [50], hydrogen sulfide [51], or ethylene [52].

Pattern formation speed is relevant to producing spatial structures in time scales allowed by experiments. This speed is reflected by the magnitude of *Re*(λ) > 0, that determines how fast unstable solutions diverge from the fixed point. Although the high dimensionality of our model makes it challenging to derive analytical expressions for the pattern formation speed (as was done in [30, 53] for two-dimensional models), computing dispersion relations for different parameters demonstrates that pattern formation speed around the nominal parameter set is inversely related to *α*_3_ (Fig 2b), but proportional to *D* (S1 Fig), *α*_1_ and *P*_*m*_ (S2 Fig). Although pattern formation speed will typically be very slow near the transition to Turing instability, our simulations within the Turing parameter space demonstrate formation of patterns within two hours (Fig 3c and 3d), making the system quite accessible to experimental observation.

Bacteria engineered with this gene circuit could be tested on agar plates or inside microfluidic chambers, both of which are amenable to spatiotemporal data collection with an epifluorescence microscope. In one spatial dimension, one could use microfluidic devices like the long trap [26], or an annular trap [54] to study periodic boundary conditions. Agar could be patterned into one-dimensional spatial structures, but these are likely to exhaust nutrients or lose moisture too quickly. Spatial patterns in two dimensions are easier to generate on agar plates (as in [5, 23, 24]), because use of microfluidics would likely be complicated by diffusional anisotropies resulting from constant perfusion of liquid media through the channels. If this were not a challenge then one could exploit the gas-permeability of polydimethylsiloxane (commonly used to fabricate microfluidic devices) to allow for selective diffusion of H_2_O_2_ (as in [27]), or sequester the gas using vacuum channel in the microfluidic device (another use for the vacuum channel that was originally built for shear-free loading of cells [55]).

Another potential challenge to implementing these results experimentally arises from the assumptions made about diffusion. First, transport through and between the cells is only approximated by the diffusion equation. Limited membrane permeability and cell crowding can slow transport of signaling molecules, resulting in a smaller effective diffusion coefficient [56]. The effective diffusion coefficients of AHL and H_2_O_2_ have not been measured directly for bacterial monolayers in microfluidic devices. However, Danino and colleagues [26] measured conduction velocity of AHL-mediated activation to be *V* = 8.5-35 $\frac{\mu m}{min}$, which combined with the model prediction of *V* = 0.17 $D_{L}^{1/2}$, results in *D*_*L*_ = 2.5-42 × 10^3^ $\frac{\mu m^{2}}{min}$. This estimate is comparable to *D*_*L*_ = 5.9-29 × 10^3^ $\frac{\mu m^{2}}{min}$ (lower limit for biofilm; upper limit for water at 25°C) estimated by Stewart [56]. For H_2_O_2_, *D*_*H*_ = 72-120 × 10^3^ $\frac{\mu m^{2}}{min}$ [56], implying that the diffusion ratio, *D* = 4-12. However, Prindle *e*t al. measured H_2_O_2_-mediated fluorescence conduction velocity *V* ≈ 700 $\frac{\mu m}{min}$ across the biopixels microfluidic device [27], so using the 0.17 prefactor that Danino [26] found demonstrates that the ratio, *D* = 100, used in our model is reasonable. In addition, decreasing *D* as much as fourfold from *D* = 100 can still allow for patterning to occur (S1 Fig). If required, further disparity between gas and lactone diffusion could be attained by using crowding agents like polyethylene glycol, or engineering circuits with slower diffusing quorum sensing molecules [48].

The mathematical model presented could be further investigated by incorporating several additional layers of detail. This includes a more biophysical description of AHL production (LuxI catalyzes the conversion of SAM to AHL), as well as H_2_O_2_ production (NDH-2 catalyzes formation of H_2_O_2_), and regulation of the *aiiA* promoter (for example, H_2_O_2_ oxidizes ArcA to relieve repression of *lux* promoter [27]). All of these processes have been lumped together (represented by dashed arrows in Fig 1) in the current model, but may turn out to have a non-negligible effect on dynamics. Our model also lumps the processes transcription, translation and protein maturation. Thus it would also be interesting to include these processes and also compare this system to one reduced to a spatially-extended delay differential equation. Previous work [57, 58] suggested that delays can predispose that system to oscillatory patterning, which is consistent with what we found upon the inclusion of slower AiiA dynamics (Figs 2a and 4a). Another feature that the current model ignores is the effect of protein dilution due to cell growth. While this may hinder implementation in exponentially growing cells, there is also work suggesting that an expanding domain can help make the Turing patterning regime more robust [57]. Furthermore, the dilution problem can be fundamentally resolved by engineering the chemical reactions in an *in vitro* transcription-translation system [59].

Considering that protein numbers are low at the steady states analyzed (the nanomolar range corresponds to tens of molecules per *E. coli*), it would be prudent to further examine this system using stochastic simulations. Hsia and colleagues [29] found that such stochastic effects changed the patterning parameter parameter space, but did not destroy the ability to produce Turing patterning. Although external fluctuations tend to destabilize the Turing parameter regime in a continuum setting [57], it has also been shown that when space is discrete, either intrinsic or extrinsic noise can cause patterning over larger parameter ranges [60, 61]. Testing these additional considerations is a good direction for future modeling studies.

Despite these theoretical and experimental challenges in further studying the proposed circuit we believe it is well worth the effort, as these types of gas and quorum sensing circuits could be quite useful for studies of bacterial morphogenesis and several synthetic biology applications. The present work provides a conceptual foundation and guidance for the development of gas mediated spatial patterning in multi-cellular and cell-free gene expression systems.

## Supporting Information

S1 Table Nominal parameter set. (PDF)

S1 Fig Effects of the diffusion coefficients ratio on the eigenvalue-wavenumber curves. Dependence of the dispersion relation on the ratio of diffusion coefficients, *D*. (a) At the nominal parameter set (S1 Table) except with *α*_1_ = 2.5 and *α*_3_ = 1.5, the Turing instability persists for *D* ≥ 45. (b) For *α*_3_ = 2.5, despite a Hopf instability at *k* = 0, Turing patterns are maintained when decreasing the ratio as low as *D* = 25.
(PDF)

S2 Fig Effect of LuxI and LuxR parameter on the eigenvalue-wavenumber curves. Dependence of the dispersion relation on LuxI and LuxR parameters. Varying these parameters around the nominal parameter set (S1 Table) demonstrates that the pattern formation speed is proportional to both: (a) LuxI maximal production (*α*_1_), and (b) amount of LuxR (*P*_*m*_).
(PDF)

S1 Equations The model incorporating the crosstalk between H_2_O_2_ and the p_*lux*_-like promoters. (PDF)

S3 Fig Effects of H_2_O_2_ crosstalk with p_*lux*_ on the eigenvalue-wavenumber curves. Dispersion relations for the expanded model (S1 Equations), varying the relative effect H_2_O_2_ on p_*lux*_, *β*. At the nominal parameter set (S1 Table) patterning still occurs with H_2_O_2_-p_*lux*_ crosstalk as high as 20%.
(PDF)

S1 Movie Spot pattern formation with no-flux boundary conditions. (MOV)
