# Correction: Turing Patterning Using Gene Circuits with Gas-Induced Degradation of Quorum Sensing Molecules

**DOI:** 10.1371/journal.pone.0160272

**Published:** 2016-07-28

**Authors:** Bartłomiej Borek, Jeff Hasty, Lev Tsimring

There is an error in Fig 2 of the published article. Panel (a) was replaced with a copy of Fig 1.

The Supporting Information file S1 Equations contains the incorrect set of differential equations.

Please see corrected Fig 2 and S1 Equations below.

**Fig 2:**
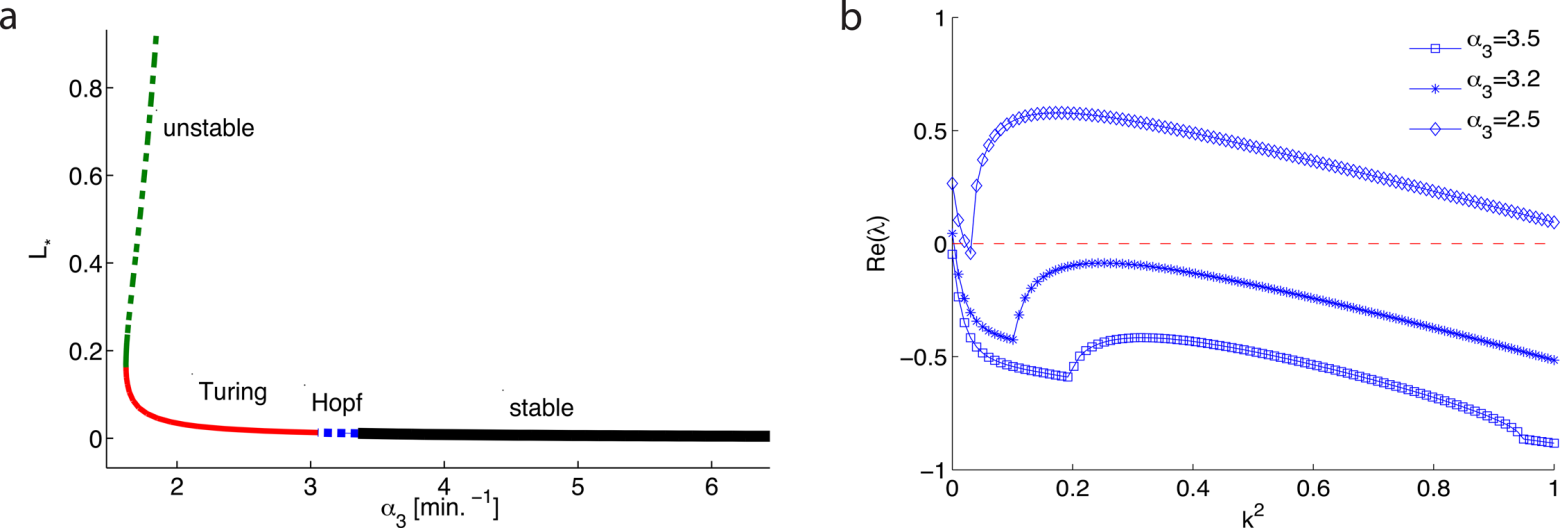
Slowing AiiA production in Eqs (1)–(4) leads to oscillations and Turing patterns. (a) A codimension one bifurcation of the AHL fixed point, L*, losing stability through a Hopf bifurcation and into a Turing instability as Aiia production rate is decreased. (b) Eigenvalue-wavenumber curves at various AiiA maximal production rates, corroborating the bifurcation analysis results. At the cusp of each curve the eigenvalues become a complex conjugate pair, with each low eigenvalue left off the plot for clarity.

## Supporting Information

S1 Equations The model incorporating crosstalk between H_2_O_2_ and the plux-like promoters. (PDF)
