# Supplementary material for: Turing Patterning Using Gene Circuits with Gas-Induced Degradation of Quorum Sensing Molecules
Source: PLoS One. 2016 May 5;11(5):e0153679. doi: 10.1371/journal.pone.0153679 (PMC4858293; doi:10.1371/journal.pone.0153679)
Supplement: S1 Table — (PDF) [file pone.0153679.s001.pdf]

Parameter	Value
α_1	4.5
α_2	1.667
α_3	3.0
γ_1	3.0
γ_2	2.0
γ_3	1.1
δ_1	0.04
δ_2	0.0202
δ_3	0.0
k_{PL}	0.675
k_{LD}	0.01
k_{PH}	0.675
k_{HA}	0.675
k_1	100
k_2	248.5
P_m	1.0
D	100
