# Supplementary figures and images for: Turing Patterning Using Gene Circuits with Gas-Induced Degradation of Quorum Sensing Molecules
Source: PLoS One. 2016 May 5;11(5):e0153679. doi: 10.1371/journal.pone.0153679 (PMC4858293; doi:10.1371/journal.pone.0153679)

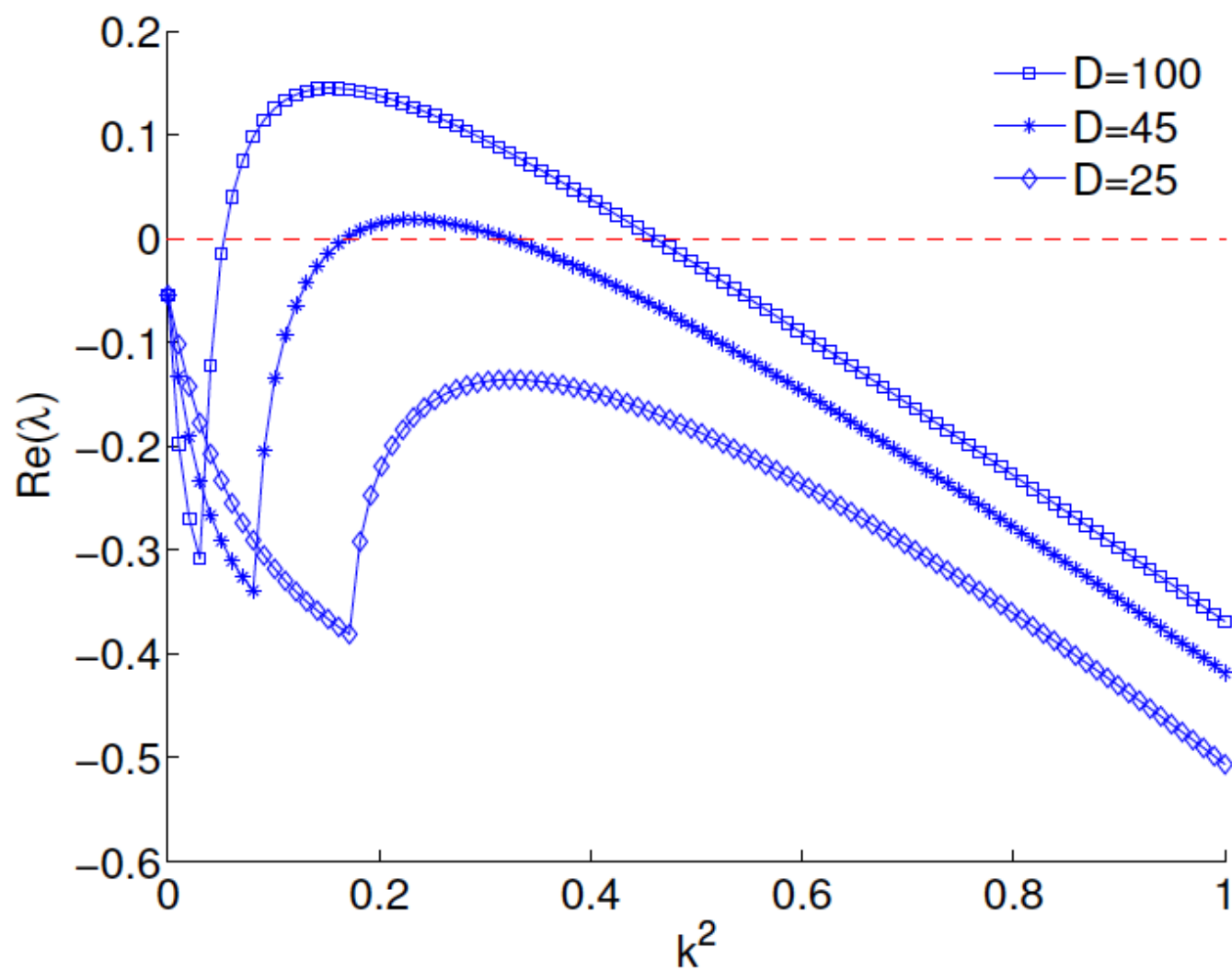

(a)

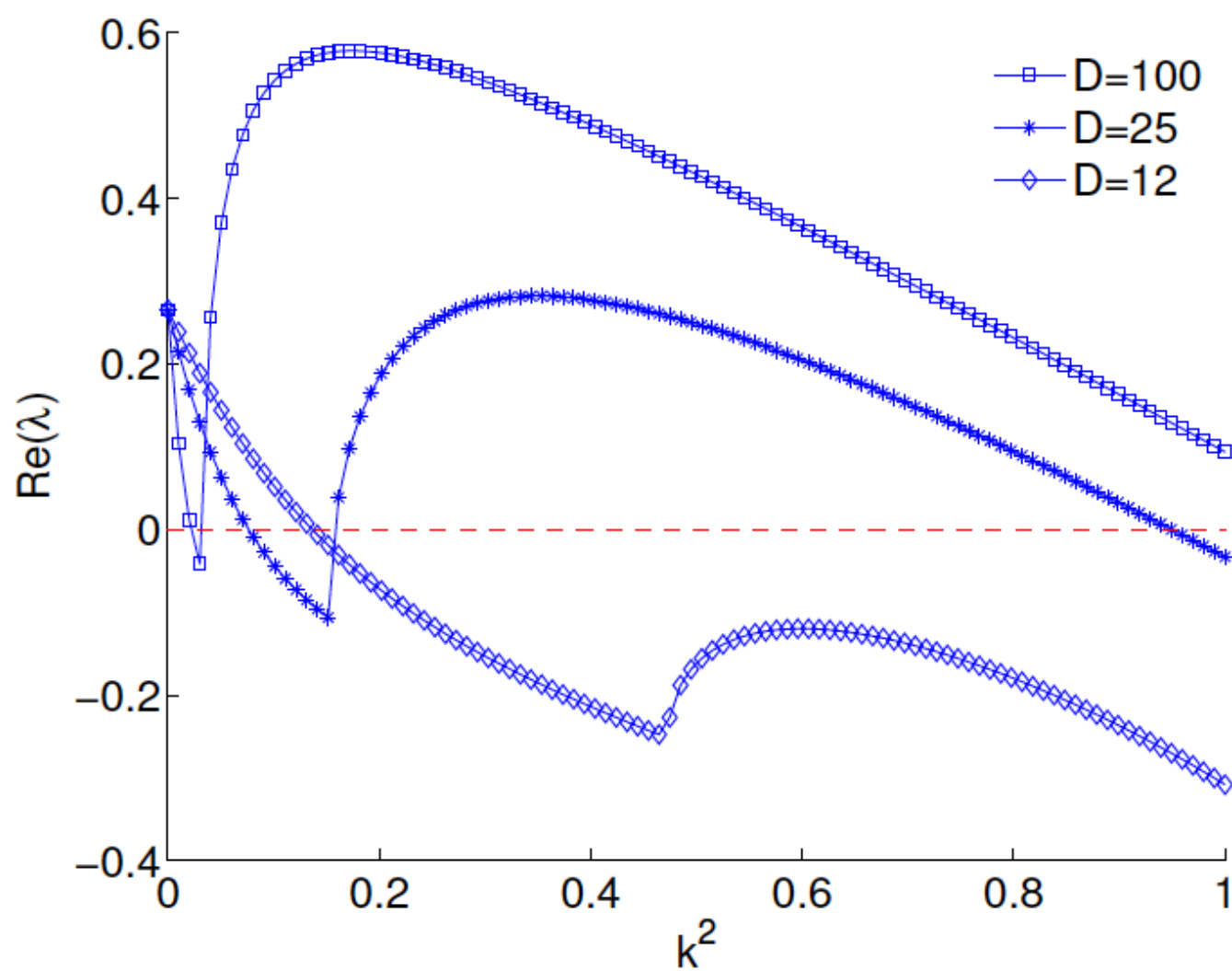

(b)

Supplement: S1 Fig — Dependence of the dispersion relation on the ratio of diffusion coefficients, D. (a) At the nominal parameter set (S1 Table) except with α1 = 2.5 and α3 = 1.5, the Turing instability persists for D ≥ 45. (b) For α3 = 2.5, despite a Hopf instability at k = 0, Turing patterns are maintained when decreasing the ratio as low as D = 25. (PDF) [file pone.0153679.s002.pdf]

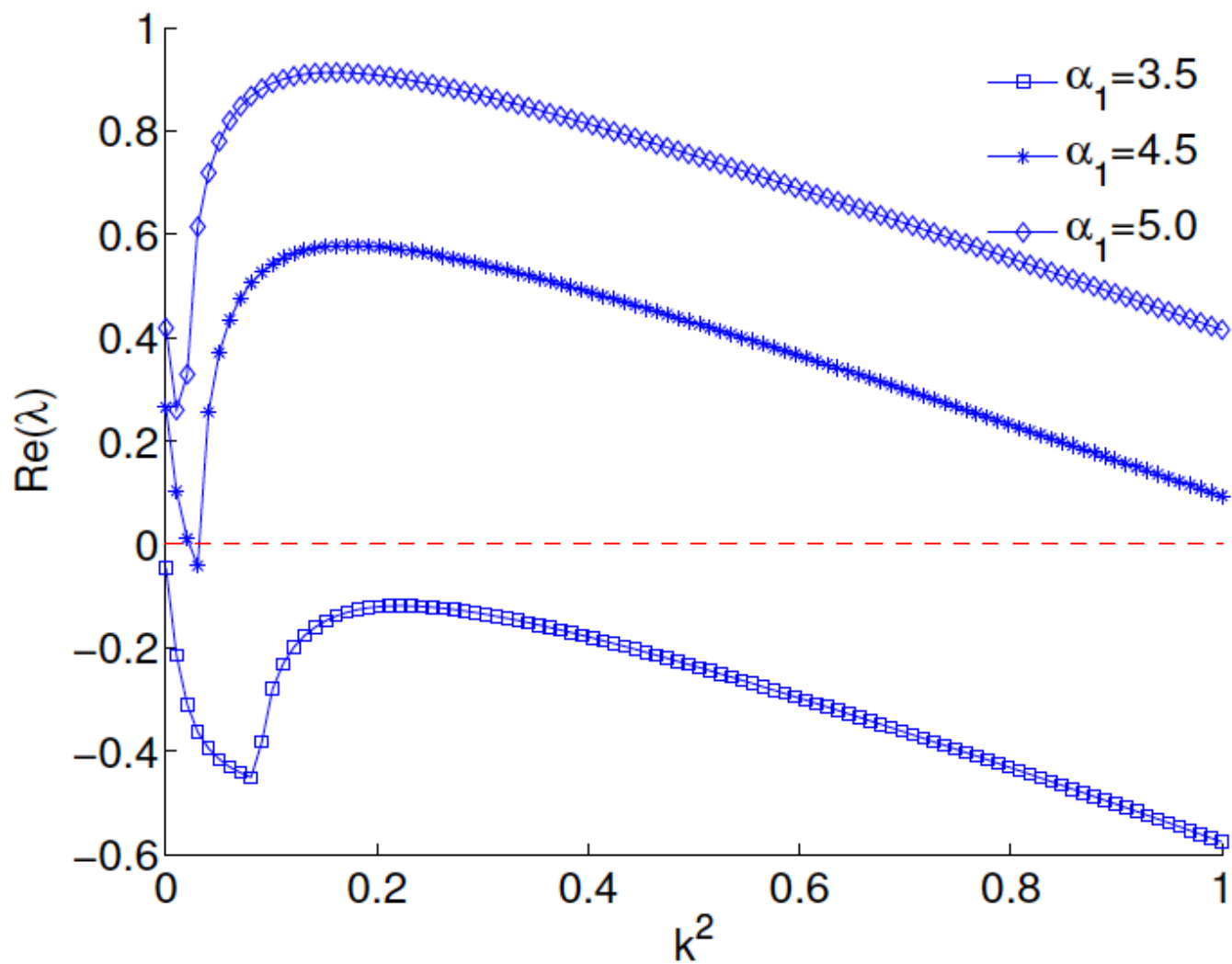

(a)

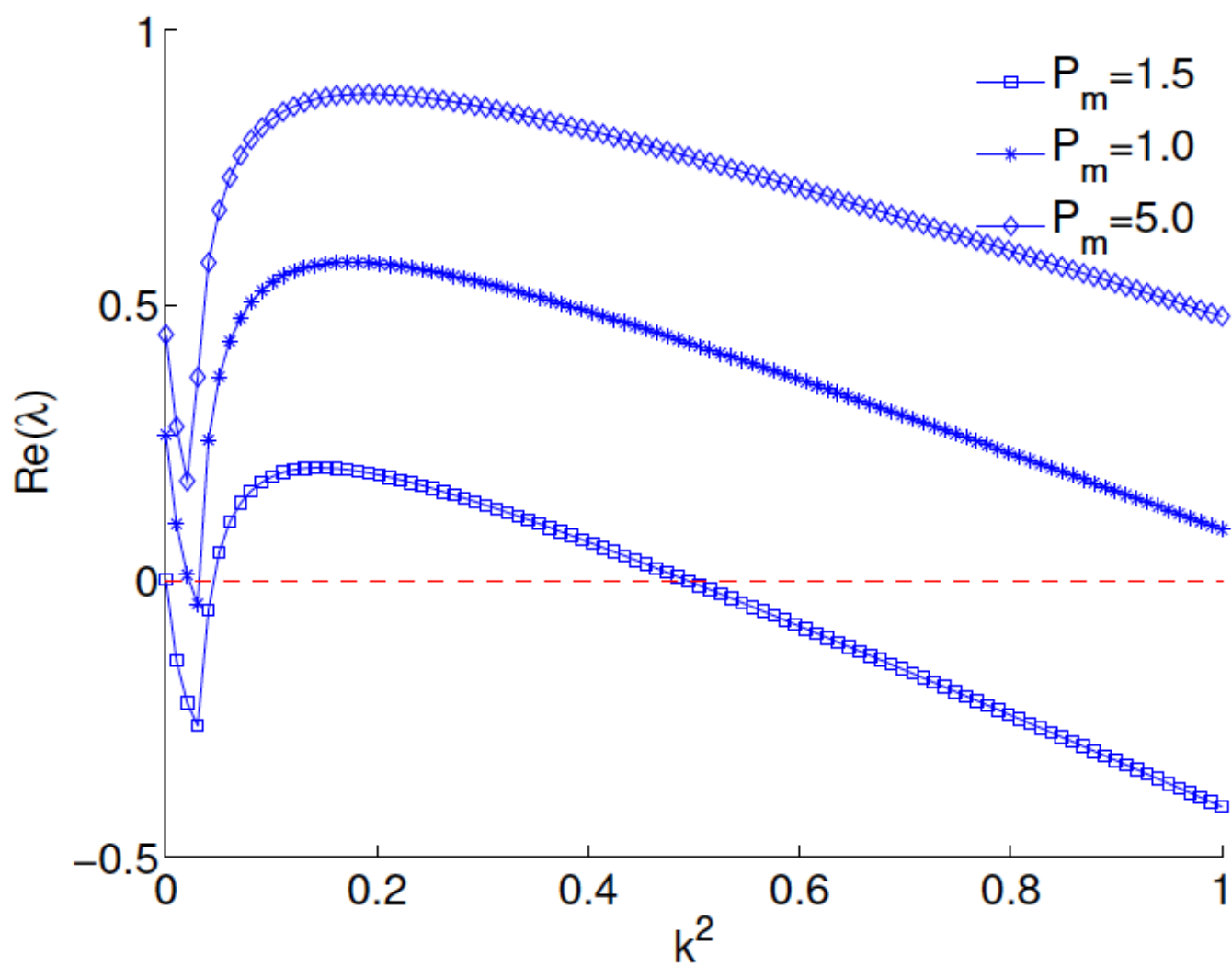

(b)

Supplement: S2 Fig — Dependence of the dispersion relation on LuxI and LuxR parameters. Varying these parameters around the nominal parameter set (S1 Table) demonstrates that the pattern formation speed is proportional to both: (a) LuxI maximal production (α1), and (b) amount of LuxR (Pm). (PDF) [file pone.0153679.s003.pdf]

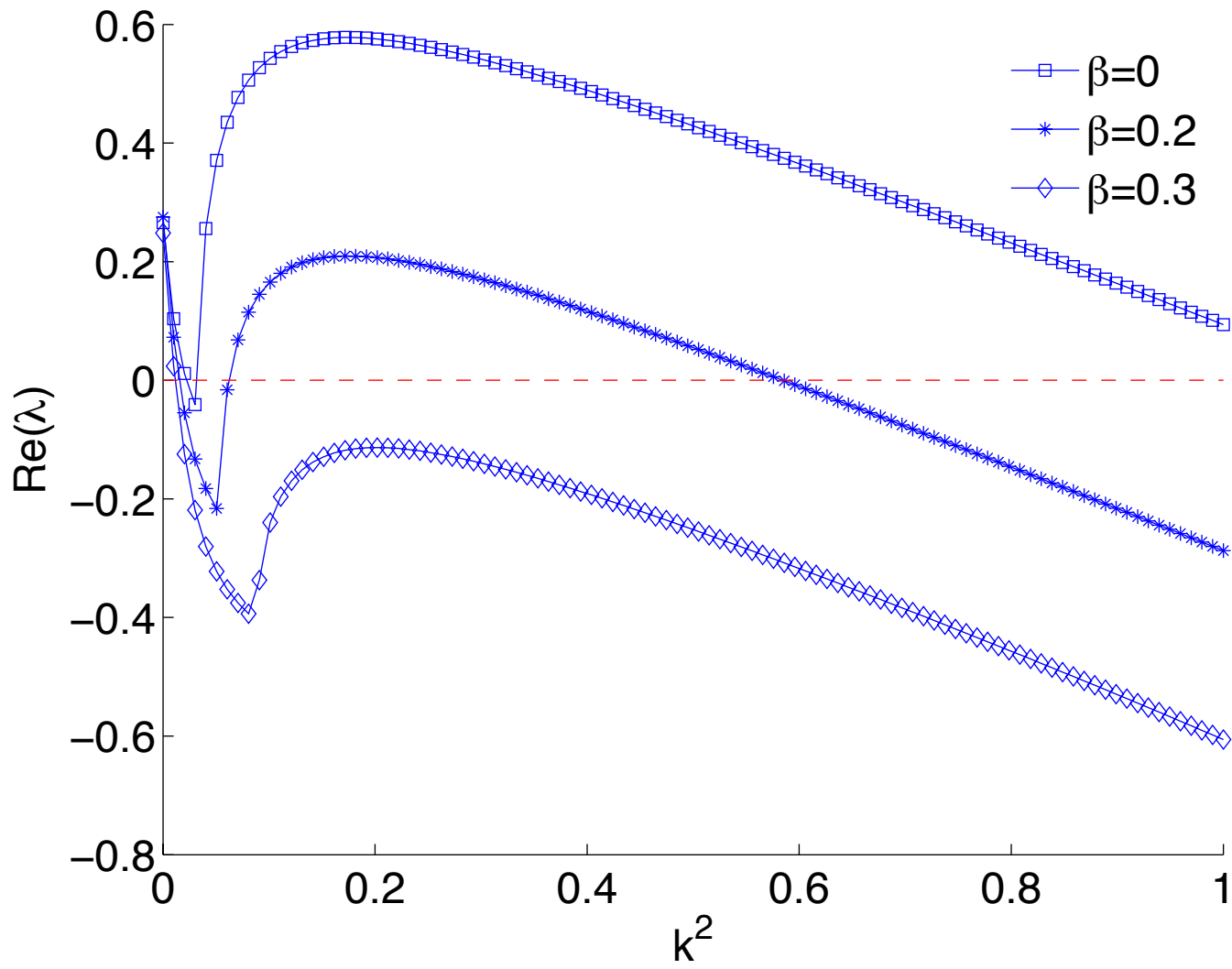

Supplement: S3 Fig — Dispersion relations for the expanded model (S1 Equations), varying the relative effect H2O2 on plux, β. At the nominal parameter set (S1 Table) patterning still occurs with H2O2-plux crosstalk as high as 20%. (PDF) [file pone.0153679.s005.pdf]
