# Supplementary material for: Turing Patterning Using Gene Circuits with Gas-Induced Degradation of Quorum Sensing Molecules
Source: PLoS One. 2016 May 5;11(5):e0153679. doi: 10.1371/journal.pone.0153679 (PMC4858293; doi:10.1371/journal.pone.0153679)
Supplement: S1 Equations — (PDF) [file pone.0153679.s004.pdf]

$$\partial_t L = \alpha_1 (\frac{\delta_1 + Q}{k_{PL} + Q}) - \gamma_1 A \frac{L}{k_{LD} + L} - k_1 (P_m - P) L + k_2 P + \nabla^2 L \tag{1}$$

$$\partial_t P = k_1 (P_m - P) L - k_2 P - 2 k_3 P^2 + 2 k_4 Q \tag{2}$$

$$\partial_t Q = k_3 P^2 - k_4 Q \tag{3}$$

$$\partial_t H = \alpha_2 (\frac{\delta_2 + Q}{k_{PH} + Q}) - \gamma_2 H + D \nabla^2 H \tag{4}$$

$$\partial_t A = \alpha_3 \frac{\delta_3 + H}{k_{HA} + H} - \gamma_3 A \tag{5}$$
