# Supplementary material for: Correction: Turing Patterning Using Gene Circuits with Gas-Induced Degradation of Quorum Sensing Molecules
Source: PLoS One. 2016 Jul 28;11(7):e0160272. doi: 10.1371/journal.pone.0160272 (PMC4965048; doi:10.1371/journal.pone.0160272)
Supplement: S1 Equations — (PDF) [file pone.0160272.s001.pdf]

$$\partial_t L = \alpha_1 (\frac{\delta_1 + P + \beta H}{k_{PL} + P + \beta H}) - \gamma_1 A \frac{L}{k_{LD} + L} - k_1 (P_m - P) L + k_2 P + \nabla^2 L$$

$$\partial_t P = k_1 (P_m - P) L - k_2 P$$

$$\partial_t H = \alpha_2 (\frac{\delta_2 + P + \beta H}{k_{PH} + P + \beta H}) - \gamma_2 H + D \nabla^2 H$$

$$\partial_t A = \alpha_3 \frac{\delta_3 + H}{k_{HA} + H} - \gamma_3 A$$
